# Tuberculosis in HIV-Negative and HIV-Infected Patients in a Low-Incidence Country: Clinical Characteristics and Treatment Outcomes

**DOI:** 10.1371/journal.pone.0034186

**Published:** 2012-03-30

**Authors:** Lukas Fenner, Sebastien Gagneux, Jean-Paul Janssens, Jan Fehr, Matthias Cavassini, Matthias Hoffmann, Enos Bernasconi, Jacques Schrenzel, Thomas Bodmer, Erik C. Böttger, Peter Helbling, Matthias Egger, for the Swiss HIV Cohort and Molecular Epidemiology of Tuberculosis Study Groups

**Affiliations:** 1 Institute of Social and Preventive Medicine (ISPM), University of Bern, Bern, Switzerland; 2 Department of Medical Parasitology and Infection Biology, Swiss Tropical and Public Health Institute, Basel, Switzerland; 3 University of Basel, Basel, Switzerland; 4 Division of Pneumology, University Hospital Geneva, Geneva, Switzerland; 5 Division of Infectious Diseases, University Hospital Zürich, Zürich, Switzerland; 6 Division of Infectious Diseases, University Hospital Lausanne, Lausanne, Switzerland; 7 Division of Infectious Diseases, Kantonsspital St.Gallen, St. Gallen, Switzerland; 8 Division of Infectious Diseases, Ospedale Regionale, Lugano, Switzerland; 9 Laboratory of Bacteriology, University Hospital of Geneva, Geneva, Switzerland; 10 Mycobacteriology Unit, Institute for Infectious Diseases, University of Bern, Bern, Switzerland; 11 Institute of Medical Microbiology, National Center for Mycobacteria, University of Zürich, Zürich, Switzerland; 12 Division of Communicable Diseases, Federal Office of Public Health, Bern, Switzerland; National Institute of Allergy and Infectious Diseases, United States of America

## Abstract

**Background:**

In Switzerland and other developed countries, the number of tuberculosis (TB) cases has been decreasing for decades, but HIV-infected patients and migrants remain risk groups. The aim of this study was to compare characteristics of TB in HIV-negative and HIV-infected patients diagnosed in Switzerland, and between coinfected patients enrolled and not enrolled in the national Swiss HIV Cohort Study (SHCS).

**Methods and Findings:**

All patients diagnosed with culture-confirmed TB in the SHCS and a random sample of culture-confirmed cases reported to the national TB registry 2000–2008 were included. Outcomes were assessed in HIV-infected patients and considered successful in case of cure or treatment completion. Ninety-three SHCS patients and 288 patients selected randomly from 4221 registered patients were analyzed. The registry sample included 10 (3.5%) coinfected patients not enrolled in the SHCS: the estimated number of HIV-infected patients not enrolled in the SHCS but reported to the registry 2000–2008 was 146 (95% CI 122–173). Coinfected patients were more likely to be from sub-Saharan Africa (51.5% versus 15.8%, *P*<0.0001) and to present disseminated disease (23.9% vs. 3.4%, *P*<0.0001) than HIV-negative patients. Coinfected patients not enrolled in the SHCS were asylum seekers or migrant workers, with lower CD4 cell counts at TB diagnosis (median CD4 count 79 cells/µL compared to 149 cells/µL among SHCS patients, *P* = 0.07). There were 6 patients (60.0%) with successful outcomes compared to 82 (88.2%) patients in the SHCS (*P* = 0.023).

**Conclusions:**

The clinical presentation of coinfected patients differed from HIV-negative TB patients. The number of HIV-infected patients diagnosed with TB outside the SHCS is similar to the number diagnosed within the cohort but outcomes are poorer in patients not followed up in the national cohort. Special efforts are required to address the needs of this vulnerable population.

## Introduction

Tuberculosis (TB) in patients co-infected with HIV is a major global public health challenge [1]. HIV infection is the most important factor increasing the risk of progression from latent infection with *Mycobacterium tuberculosis* to clinically active disease [2]. On the other hand, TB may induce retroviral replication [3]. Highly active antiretroviral combination therapy (ART) has substantially improved the prognosis of HIV infection and reduced the risk of TB both in industrialized and low-income countries. Nevertheless, in many resource-constrained settings TB remains the most common acquired immune deficiency syndrome (AIDS)-defining illness [4].

In Switzerland, the total number of TB cases has been decreasing for decades, but HIV-infected patients and migrants remain risk groups [5], [6]. HIV coinfection is not included in the national TB surveillance system, and can only be estimated by the number of AIDS-defining TB cases [7], [8]. Risk factors for TB in HIV-infected patients include low CD4 cell count, and the patient's origin (higher risk in patients originating from Eastern Europe, sub-Saharan Africa and Brazil compared to other patients [5]. An analysis of the Swiss HIV Cohort Study (SHCS) showed that in the ART era 142 (2.3%) of 6,160 patients had a history of TB at enrolment into the cohort, and 56 (0.9%) developed TB during follow-up [9]. In countries with a high TB burden CD4 cell count is a major risk factor for TB [10], but the risk may differ between communities [11].

In Switzerland, France, The Netherlands and other countries, HIV-infected patients are followed-up in prospective studies that have national coverage [12]–[15]. About 70% of HIV-infected patients in Switzerland with advanced disease are enrolled in the SHCS [12]. Patients included in HIV cohort studies may, however, not be representative of all patients living with HIV in the country. In particular, little is known about HIV-infected TB patients who are not enrolled in cohort studies, and about HIV-infected compared to HIV-negative TB patients.

The aim of this study was to describe the patient characteristics and treatment outcomes in HIV-infected TB patients enrolled and not enrolled in the national Swiss cohort, and to compare them to HIV-negative TB patients.

## Methods

The Swiss Molecular Epidemiology of Tuberculosis (SMET) study is a collaborative project [16] between the SHCS, the National Center for Mycobacteria, diagnostic microbiology laboratories, departments of respiratory medicine and public health, and the Federal Office of Public Health (FOPH). The overarching aim was to examine the genetic population structure of *M. tuberculosis* and the associations between strain variation, patient origin and clinical characteristics in HIV-infected compared to HIV-negative TB patients. Further information on the SMET project is available at www.tb-network.ch. All participating sites are listed in the File S1. The SHCS is a prospective observational study of HIV-infected individuals undertaken in HIV outpatient clinics in Switzerland, which has been described in detail elsewhere [14].

All HIV-infected patients diagnosed with TB in the SHCS between 2000 and 2008 whose *M. tuberculosis* complex (MTBC) isolate was available were retrospectively included in the SMET study. Both “incident” and “prevalent” TB cases were eligible, i.e. patients who developed TB during follow-up and patients diagnosed with TB at enrolment into the SHCS [9]. For each eligible SHCS patient three culture-confirmed TB cases not enrolled in the SHCS but reported to the National TB Registry during the same time period were randomly selected (288 from the reported 4,221 culture-confirmed TB cases). Notification of all TB cases in Switzerland, including information on age, sex, date of birth, geographic origin, legal status, drug resistance is mandatory in Switzerland, but TB treatment outcomes are not reported.

In HIV-infected patients additional information was obtained using a standardized questionnaire that was sent to the treating physicians, including information on TB treatment and outcomes. TB treatment outcomes were defined according to international standards [17]–[19]. Treatment outcomes were further grouped into three categories: “successful” included cure and treatment completion; “unsatisfactory” included treatment failure, treatment interruption, transfer-out and unknown; and “death” of any cause while on treatment [17]. Drug resistance was defined as any resistance to isoniazid, rifampicin or ethambutol as reported to the TB registry.

Differences between groups in binary variables were tested using χ2 tests or Fisher's exact tests, continuous variables by Wilcoxon rank sum test. Binomial 95% confidence intervals (CI) were calculated where appropriate. All analyses were performed in Stata version 11.1 (Stata Corporation, College Station, TX).

The study was approved by the Ethics Committee of the University of Bern, Switzerland. Written informed consent was obtained from all patients enrolled in the SHCS. For patients outside the SHCS, informed consent was obtained by the treating physicians. In some cases informed consent could not be obtained from the patient because he or she could not be located or was known to have died. For these cases we obtained permission from the Federal expert commission on confidentiality in medical research to use the data provided by the treating physician.

## Results

In the SHCS 144 definite (culture-confirmed) TB cases were diagnosed during the study period 2000–2008. Among these, 93 cases (64.6%) had an MTBC isolate available from the strain collections and were included in the study. The TB registry included 4,221 culture-confirmed cases. A total of 288 cases were randomly selected (approximately three cases for every SHCS case with an available MTBC isolate). The sample included 10 (3.5%) HIV-infected patients not enrolled in the SHCS. Based on the proportion of HIV-infected patients in the randomly selected registry sample and its binomial confidence interval (2.9%–4.1%), it can be estimated that 146 (95% CI 122–173) HIV-infected patients not enrolled in the SHCS were among the TB cases reported to the registry 2000–2008.

Data on a total of 381 TB patients were analyzed, including 103 (26.3%) HIV-infected patients. Table 1 compares socio-demographic and clinical characteristics between HIV-infected and HIV-negative TB patients, and of HIV-infected patients enrolled and not enrolled in the SHCS. The groups were similar regarding age and sex, but HIV-infected patients were less likely to have cavitary disease and more likely to have disseminated disease than HIV-negative patients. HIV-infected patients were also more likely to have been born in sub-Saharan Africa (51.5% versus 15.8%, p<0.0001). The proportion of patients with multidrug resistance was higher in HIV-infected patients compared to HIV-negative patients (1.9% versus 0%) but this failed to reach conventional statistical significance (p = 0.07 by Fisher's exact test).

**Table 1:** Comparisons of patient characteristics between HIV-infected and HIV-negative tuberculosis patients, and HIV-infected tuberculosis patients enrolled and not enrolled in the Swiss HIV Cohort Study (SHCS).

Characteristic	HIV-infected patients (n = 103)			HIV-negative patients (n = 278)	*P* values	
	All	Non-SHCS (n = 10)	SHCS (n = 93)	All	HIV-infected versus HIV-negative	SHCS versus non-SHCS
Age, median (IQR), years	36 (32–45)	33 (28–41)	37 (33–45)	41 (28–63)	0.037	0.21
Male sex, n (%)	49 (47.6)	5 (50.0)	44 (47.3)	139 (50.0)	0.67	0.87
Cavitary disease, n (%)	11 (10.7)	0 (0)	11 (11.8)	62 (22.3)	0.010	0.25
Clinical manifestation, n (%)						
Pulmonary	71 (68.9)	7 (70.0)	64 (68.8)	205 (73.7)	0.35	1.0^3^
Extra-pulmonary	32 (31.1)	3 (30.0)	29 (31.2)	73 (26.3)	0.35	1.0^3^
Disseminated	24 (23.3)	5 (50.0)	19 (20.4)	13 (4.7)	<0.0001	0.050^3^
Birth origin, n (%)					<0.0001	1.0^3^
Switzerland	25 (24.3)	1 (10.0)	24 (25.8)	70 (25.2)		
Europe (without Switzerland)	10 (9.7)	1 (10.0)	9 (9.7)	75 (27.0)		
Asia	11 (10.7)	1 (10.0)	10 (10.8)	66 (23.7)		
Sub-Saharan Africa	53 (51.5)	5 (50.0)	48 (51.6)	44 (15.8)		
Central/South America	3 (2.9)	2 (20.0)	1 (1.1)	10 (3.6)		
Other regions/unknown	1 (1.0)	0	1 (1.1)	13 (4.7)		
Any drug resistance 1, n (%)	11 (10.7)	1 (10.0)	10 (10.8)	17 (6.1)	0.13	1.0^3^
Multidrug resistance, n (%)	2 (1.9)	0	2 (2.2)	0 (0)	0.073^c^	1.0^3^
CD4 cell count 2, median (IQR), cells/µl	148 (66–280)	79 (14–203)	149 (72–284)	-	-	0.071
HIV RNA viral load 2, median (IQR), copies/ml	112,800 (11,400–295,000)	196,813 (24,900–327000)	99,503 (7,050–259,000)	-	-	0.46

95% CI, 95% confidence interval; IQR, interquartile range.

1 Any drug resistance to isoniazid, rifampicin or ethambutol as reported to the Federal Office of Public Health.

2 At the time of TB diagnosis.

3 Fisher's exact test.

Among HIV-infected TB patients the most likely sources of HIV infection were heterosexual transmission (59, 57.3%), injecting drug use (13, 12.6%) and men having sex with men (10, 9.7%). HIV-infected TB patients were most frequently from sub-Saharan Africa. Median time since immigration from sub-Saharan Africa until TB diagnosis was 3.3 years (interquartile range [IQR] 0.8–5.6). The 10 patients not enrolled in the SHCS included four asylum seekers or refugees, two migrant workers, three other foreigners (visitors, students, etc.), and one patient with unknown legal status. Four of the 10 patients were diagnosed in Geneva. Patients not enrolled in the cohort were younger than SHCS patients and had lower CD4 cell counts at TB diagnosis (median CD4 cell count 79 cells/µL versus 149 cells/µL, p = 0.07, Table 1). Of the 93 SHCS patients, 36 (38.7%) were diagnosed with TB while on ART. ART was initiated in all patients, with a median delay of 5 weeks (IQR 0–12.9) after TB diagnosis among those not yet on ART.

**Figure 1:**
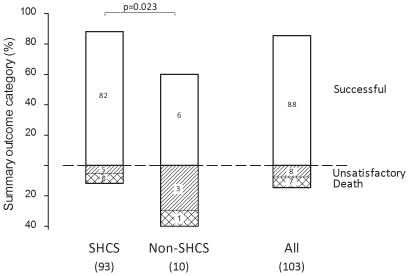
Summary categories of treatment outcomes in HIV-infected TB patients enrolled in the Swiss HIV Cohort Study (SHCS) and not enrolled in the SHCS (non-SHCS). Successful outcomes included cure and treatment completed; unsatisfactory outcomes treatment failure, interruption and transfer-out. Overall, only one patient with treatment failure was reported. Bars add to 100%. *P* value from Fisher's exact tests.

Outcomes of TB treatment in the 103 HIV-infected patients are summarized in Figure 1. Overall, TB treatment was successful in 88 (85.4%) patients and unsatisfactory in 8 patients (7.8%); 7 patients (6.8%) died. A total of 39 patients (37.9%) completed treatment, in 49 (47.6%) cure was documented and five patients (4.9%) were transferred. One patient experienced treatment failure, one interrupted treatment, and the treatment outcome was unknown in another case. Treatment outcome was poorer in patients not enrolled in the SHCS: there were 6 patients (60.0%) with successful outcomes compared to 82 (88.2%) patients among SHCS participants (p = 0.023 by Fisher's exact test). Among the seven deaths, three were documented to be TB-related; the other deaths were due to other reasons or not ascertained. Fifty-six patients (54.4%) were treated with a standard treatment regimen including isoniazid (H), rifampicin (R), ethambutol (E), and pyrazinamide (Z); H/Z/E plus Rifabutin was used in 19 cases (18.4%), and H/R/Z/E plus one additional antituberculosis drug was used in 10 cases (9.7%), and in 18 (17.5%) cases another non-standard regimen was used.

## Discussion

In this nation-wide study of HIV-infected and HIV-negative TB patients we found that the two groups were similar with respect to age and sex, and in both groups about a quarter of patients were born in Switzerland. The geographic origin of HIV-infected patients differed: the majority of HIV-infected TB patients were from sub-Saharan Africa, the region most heavily affected by HIV. As previously described for developing and industrialized countries [20], [21], cavitary disease was less frequent and disseminated disease more frequent among HIV-infected patients.

The SHCS has national coverage, but the cohort does not appear to be representative of all HIV-infected TB patients. Our random sample from the national TB registry identified 10 HIV-infected patients not enrolled in the SHCS. It can thus be estimated that about 146 additional HIV-infected TB patients not enrolled in the SHCS were diagnosed during the study period. We found that these patients were a vulnerable, marginalized group of immigrants and asylum seekers with poor TB treatment outcomes. TB treatment outcomes among HIV-infected patients were satisfactory overall: the 85% successful treatments compare favorably with other European countries for which success rates (regardless of HIV status) of 70% to 80% have been reported [22]. In particular, transfer-outs were more frequent in the non-cohort patient group, whereas patients with treatment failure were rare (one patient only).

We found that outcomes of HIV-infected TB patients are poorer in patients not followed up in the national cohort. Our results suggest that enrolment in the SHCS may have a beneficial effect on treatment outcomes, or alternatively that patients who agreed to enroll in the SHCS may have better health and be more health conscious than other patients. This finding is consistent with a previous analysis of the SHCS which showed that patients who were seen regularly in the cohort from early stages of the infection were, at comparable levels of immunodeficiency, less likely to progress to AIDS, and mortality was reduced [23]. Similarly, a study from Birmingham, Alabama, found that patients who missed visits after initiating outpatient treatment for HIV infection were at higher risk of death compared with patients who attended all scheduled appointments [24]. A recent study from the SHCS found that non-Europeans, particularly from sub-Saharan Africa, were less likely to participate in the SHCS compared to Europeans [25].

Of note, four out of 10 HIV-infected TB patients from outside the SHCS were diagnosed in Geneva. Geneva has the highest incidence of TB in the country, which is probably explained by the relatively large number of undocumented migrants living in the area [21], [26]. Since 1996, a mobile outpatient unit has offered free health care to this population, with excellent uptake [21], [26]. There are several possible reasons why these patients were not enrolled in the SHCS after HIV infection was diagnosed: patients may be reluctant to engage with more formal health structures or may be unable to do so due to their fragile socio-economic situation, and physicians may not ask patients to enroll in the cohort because of the patients' uncertain situation or language barriers.

Our study has several limitations. We analyzed a small sample of HIV-infected TB patients from outside the SHCS only, who were identified in the random sample drawn from the national TB registry. Unfortunately, the registry cannot be linked to the mandatory, anonymous reports of HIV infections, both for practical and legal reasons. However, the non-SHCS cases were obtained from a random sample of all culture-confirmed TB cases notified to the National TB Registry during the study period which allowed us to estimate the total number of non-SHCS TB cases. In addition, we included only patients with available MTBC isolates, which excluded culture-negative TB cases. Finally, outcome analysis of TB treatment was limited to HIV-infected patients.

In conclusion, our findings indicate that in Switzerland a sizeable group of vulnerable HIV-infected migrants with TB may not receive optimal care and follow-up. Special efforts are required to address the needs of this population.

## Supporting Information

File S1 **Study group members of the Swiss HIV Cohort and Molecular Epidemiology of Tuberculosis Study Groups.**
(DOC)
