# Supplementary material for: Tuberculosis in HIV-Negative and HIV-Infected Patients in a Low-Incidence Country: Clinical Characteristics and Treatment Outcomes
Source: PLoS One. 2012 Mar 30;7(3):e34186. doi: 10.1371/journal.pone.0034186 (PMC3316631; doi:10.1371/journal.pone.0034186)
Supplement: File S1 — Study group members of the Swiss HIV Cohort and Molecular Epidemiology of Tuberculosis Study Groups. (DOC) [file pone.0034186.s001.doc]

*PLoS ONE, Revision 2, Production file – corrected 3 – supporting file*

**File S1. Study group members** **of the Swiss HIV Cohort and Molecular Epidemiology of Tuberculosis Study Groups**

**The members of the Swiss Molecular Epidemiology of Tuberculosis Study Group**

Central coordinating team:

Lukas Fenner and Matthias Egger, Institute of Social and Preventive Medicine, Bern; Sebastien Gagneux and Marcel Tanner, Swiss Tropical and Public Health Institute, Basel; Hansjakob Furrer, Inselspital Bern.

National Center for Mycobacteria:

Erik C. Böttger, Institute of Medical Microbiology, University of Zürich, Switzerland.

Microbiology laboratories:

Reno Frei, Clinical Microbiology, University Hospital Basel; Thomas Bodmer, Institute for Infectious Diseases, University of Bern; Beatrice Ninet, Jacques Schrenzel, Central Laboratory of Bacteriology, University Hospital Geneva; Katia Jaton, Amalio Telenti, Institute of Microbiology, University Hospital of Lausanne; Hans Siegrist, ADMed Microbiology, La Chaux-de-Fonds; Gaby E. Pfyffer, Department of Medical Microbiology, Luzerner Kantonsspital, Luzern; Thomas Bruderer, Centre for Laboratory Medicine, St.Gallen; Marisa Dolina, Cantonal Institute of Microbiology, Medical Bacteriology, Bellinzona; Olivier Dubuis, Viollier AG Switzerland, Allschwil.

Swiss HIV Cohort Study:

Manuel Battegay, University Hospital Basel; Enos Bernasconi, Andrea Parini Lugano; Matthias Hoffmann, St.Gallen; Hansjakob Furrer, Inselspital Bern; Matthias Cavassini, University Hospital of Lausanne; Bernard Hirschel, Alexandra Calmy, University Hospital of Geneva; Jan Fehr, University Hospital of Zürich.

Respiratory clinics:

Jean-Paul Janssens, University Hospital of Geneva; Jesica Mazza Stalder, University Hospital of Lausanne.

Federal Office of Public Health:

Peter Helbling and Ekkehardt Altpeter, Division of Communicable Diseases.

The Union:

Hans L. Rieder, Institute of Social and Preventive Medicine, University of Zürich, Switzerland; The Union, Paris, France.

**The members of the Swiss HIV Cohort Study**

Barth J, Battegay M, Bernasconi E, Böni J, Bucher HC, Burton-Jeangros C, Calmy A, Cavassini M, Cellerai C, Egger M, Elzi L, Fehr J, Fellay J, Flepp M, Francioli P (President of the SHCS), Furrer H (Chairman of the Clinical and Laboratory Committee), Fux CA, Gorgievski M, Günthard H (Chairman of the Scientific Board), Haerry D (deputy of "Positive Council"), Hasse B, Hirsch HH, Hirschel B, Hösli I, Kahlert C, Kaiser L, Keiser O, Kind C, Klimkait T, Kovari H, Ledergerber B, Martinetti G, Martinez de Tejada B, Metzner K, Müller N, Nadal D, Pantaleo G, Rauch A, Regenass S, Rickenbach M (Head of Data Center), Rudin C (Chairman of the Mother & Child Substudy), Schmid P, Schultze D, Schöni-Affolter F, Schüpbach J, Speck R, Taffé P, Tarr P, Telenti A, Trkola A, Vernazza P, Weber R, Yerly S.
